# Ingestion of genetically modified yeast symbiont reduces fitness of an insect pest via RNA interference

**DOI:** 10.1038/srep22587

**Published:** 2016-03-02

**Authors:** Katherine A. Murphy, Christine A. Tabuloc, Kevin R. Cervantes, Joanna C. Chiu

**Affiliations:** 1 Department of Entomology and Nematology, College of Agricultural and Environmental Sciences, University of California, Davis, CA 95616.

## Abstract

RNA interference has had major advances as a developing tool for pest management. In laboratory experiments, double-stranded RNA (dsRNA) is often administered to the insect by genetic modification of the crop, or synthesized *in vitro* and topically applied to the crop. Here, we engineered genetically modified yeast that express dsRNA targeting *y-Tubulin* in *Drosophila suzukii*. Our design takes advantage of the symbiotic interactions between *Drosophila*, yeast, and fruit crops. Yeast is naturally found growing on the surface of fruit crops, constitutes a major component of the *Drosophila* microbiome, and is highly attractive to *Drosophila.* Thus, this naturally attractive yeast biopesticide can deliver dsRNA to an insect pest without the need for genetic crop modification. We demonstrate that this biopesticide decreases larval survivorship, and reduces locomotor activity and reproductive fitness in adults, which are indicative of general health decline. To our knowledge, this is the first study to show that yeast can be used to deliver dsRNA to an insect pest.

In recent years, RNA interference (RNAi) has shown great potential to become a powerful tool in pest management. RNAi is effective in many economically important insects, including honey bee, aphid, termite, mosquito, western corn rootworm, and Colorado potato beetle^1^,^2^,^3^,^4^,^5^,^6^,^7^,^8^. With genomic data becoming increasingly available for non-model insects, sequence-based pest management strategies are becoming more practical. Inducing mortality through RNAi has several advantages over conventional chemical pesticides. As a consequence of its sequence-dependent mode of action, RNAi can be tailored to target only pest species and spare beneficial insects^9^,^10^. This is accomplished by choosing to target unique mRNA sequences within the pest species. Likewise, RNAi pesticides can be designed to target a broad range of insects by choosing sequences that are more conserved between species. Another advantage of RNAi pesticides is that the “active ingredient” is RNA, which is organic, biodegradable, and can be inexpensively produced within microorganisms.

RNAi is a cellular mechanism that likely evolved to protect eukaryotes from RNA viruses^11^. To activate the RNAi pathway, double stranded RNA (dsRNA) can be fed to insects and absorbed into the cells that line the midgut. Exogenous dsRNA is usually processed into 20–30 nucleotide duplexes by the ribonuclease III enzyme DICER^12^,^13^,^14^. These nucleotide duplexes are assembled onto the catalytic component ARGONAUTE and a single guide strand is incorporated into the RNA induced silencing complex (RISC)^13^. The guide strand binds to complementary mRNAs, and the RISC complex mediates degradation or translational suppression of the endogenous transcript. The RISC complex will cleave the mRNA when base pair matching is highly complementary, or alternatively, the complex can bind the mRNA and suppress translation when there are mismatched base pairs. Degradation or suppression of transcripts that code for critical genes in the insect results in decreased amounts of critical gene products and increased mortality^14^,^15^.

Some species, such as *C. elegans*, possess a SID-1 RNA channel transporter that facilitates uptake of dsRNA into cells^16^. Dipterans do not posses a SID1 ortholog, but are able to absorb dsRNA through other mechanisms, such as receptor mediated endocytosis^17^. Studies have shown dsRNA can enter *Drosophila melanogaster* cells through injection into whole animals^18^ or through addition to the growth medium of cultured cells^19^. Whyard *et al.*^20^ demonstrated that Drosophilids are able to uptake dsRNA from oral ingestion of dsRNA solution containing a lipid encapsulating reagent, resulting in tissue-specific gene suppression and mortality. Importantly, this study supports the idea that a Drosophilid pest could be controlled through oral delivery of dsRNA.

Delivery of intact dsRNA into insect cells remains a challenge, although many delivery methods have been developed^14^. Crops can be genetically engineered to express dsRNA targeting insect pests^6^,^21^. The use of modified plants has proven remarkably effective in managing coleopteran pests such as western corn rootworm^6^. Another delivery method is to synthesize the dsRNA *in vitro*, and then apply to foliage by spraying^22^, or to roots by soaking^23^, resulting in a transient presence of dsRNA within the plant tissue. Nanoparticles composed of chitosan and dsRNA have been used to deliver dsRNA to larval mosquitos through oral ingestion^24^. Zhu *et al.*^26^ demonstrated that bacteria expressing dsRNA can be fed to insects to induce RNAi^25^,^26^, and genetically modified bacteria can even colonize the gut and deliver dsRNA from within the host^27^.

The aim of this study is to demonstrate that genetically modified yeast can be used to produce and deliver dsRNA to *Drosophila*, resulting in altered gene expression and decreased fitness in the fly. We chose to use yeast as a delivery vehicle for dsRNA because of the symbiosis between yeast, *Drosophila*, and fruits. Yeast is an important endosymbiont and a major component of the microbiome in *Drosophila*^28^. Yeasts are naturally found growing on the surface of intact and rotting fruits and produce volatiles that are highly attractive to Drosophilids^29^,^30^. Palanca *et al.* quantified the attractive properties of wild yeast isolates and found fruit-associated isolates were more attractive than those not associated with fruit, regardless of taxonomic positioning^31^. Additionally, they found that *Saccharomyces cerevisiae* was the most attractive to *Drosophila melanogaster*. We therefore selected to modify *S. cerevisiae* for this study. *S. cerevisiae* lacks a functional RNAi mechanism^32^, which might make it a suitable dsRNA delivery vehicle if the absence of RNAi machinery promotes stability of dsRNA. Due to its frequent use as a model organism, *S. cerevisiae* has the added advantage of easy genetic manipulation. *Drosophila* acquire yeast from the environment and can transmit yeast horizontally to mates^33^. *Drosophila*-associated yeast spores can be passed intact through the gut and deposited onto food sources^34^, where they can be ingested by larvae or adults. Thus, yeast is a chemoattractive, transmittable symbiont of *Drosophila* and fruit crops. We postulate these characteristics make yeast an ideal and novel candidate to serve as a living biopesticide.

*Drosophila suzukii*, commonly known as Spotted Wing *Drosophila*, is an invasive pest of a variety of soft skinned fruits^35^. Native to South East Asia, this pest was first identified in California in 2008. Since then, infestations have spread across the United States, Canada, and Europe^36^, resulting in an estimated 718 million dollars in crop losses annually^37^,^38^. We chose to target *D. suzukii* in this study because it is a serious economic pest and is sufficiently homologous to the model insect *D. melanogaster* to facilitate target gene selection. Our laboratory has recently sequenced the *D. suzukii* genome, which further aids in the design of dsRNAs^39^. Additionally, yeast-baited traps have been demonstrated to effectively lure *D. suzukii* in an agricultural setting^40^, and evidence suggests that *D. suzukii* feed on yeasts in the field^41^.

## Results

### Treatment of larvae with *in vitro* transcribed dsRNA reduces expression of target genes and decreases survival to adulthood

**Figure 1:**
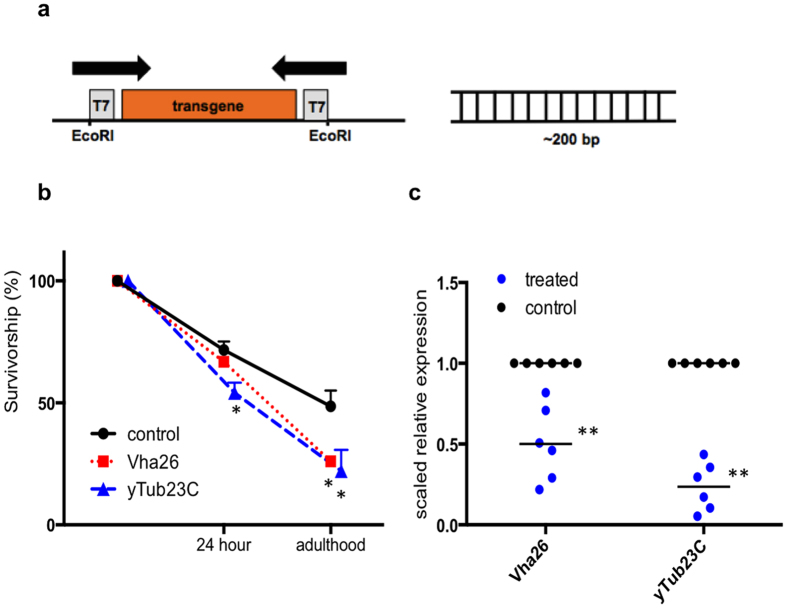
*D. suzukii* larvae treated with *in vitro* transcribed dsRNA have increased mortality and decreased target gene expression. (**a**) Map of vector for *in vitro* transcription of dsRNA using convergent T7 promoters flanking ~200 bp of *D. suzukii* target gene sequence (*yTub23C* fragment =183 bp, *Vha26 fragment* =233 bp). Black arrows indicate direction of transcription. (**b**) Survivorship of 2nd instar larvae following soaking treatment with dsRNA solution. The control treatment consisted of *Drosophila* medium and lipid encapsulating reagent. Mortality was assessed 24 hours after treatment and at the time of eclosion. A total of 160 larvae were tested in each treatment, and data represent mean and s.e.m. of three independent experiments. (**c**) Suppression of target genes *yTub23C* and *Vha26* in whole larvae 24 hours after treatment with dsRNA solution. Ten larvae were homogenized to obtain one RNA sample. Expression was quantified with RT-qPCR and normalized against housekeeping gene *Cbp20*. Each data point represents a biological replicate, and horizontal lines indicate the mean. (**b**,**c**) Asterisks represent significance as determined by two-tailed *t*-test (*indicates p < 0.05, and **indicates p < 0.01).

Previous studies have demonstrated dsRNA can be synthesized and fed to insects in solution or artificial diet, resulting in gene knockdown and mortality^1^,^20^,^42^,^43^,^44^. This method is often used to select targets for *in vivo* testing. Critical genes *tubulin* and *vacuolar ATPase* are commonly used target genes and have effectively induced mortality in Coleopterans and Dipterans^6^,^20^. First, we examined whether dsRNA targeting *y-tubulin 23C* (*yTub23C*) and *vacuolar H*+ *ATPase 26* *kD subunit* (*Vha26*) could be used to induce mortality in *D. suzukii* larvae. dsRNA was transcribed *in vitro* using a plasmid containing approximately 200 bp *D. suzukii* target gene sequence flanked with convergent T7 promoters (Fig. 1a). We found that a one-hour soaking treatment with *in vitro* transcribed dsRNA significantly reduced survival to adulthood by 54% and 46% respectively compared to the control (Fig. 1b). The control treatment had a 48% survival rate to adulthood. Next, we examined if the increased mortality was due to changes in target gene expression level. RNA was extracted from whole larval bodies 24 hours after the one-hour soaking treatment. Target transcripts *yTub23C* and *Vha26* were significantly reduced relative to the control by 76% and 50% respectively in treated samples (Fig. 1c). These results suggest dsRNA in the soaking solution is absorbed and able to trigger an RNAi response in the larvae, resulting in mortality.

### dsRNA targeting critical *D. suzukii* genes is expressed in modified *Saccharomyces cerevisiae*

**Figure 2:**
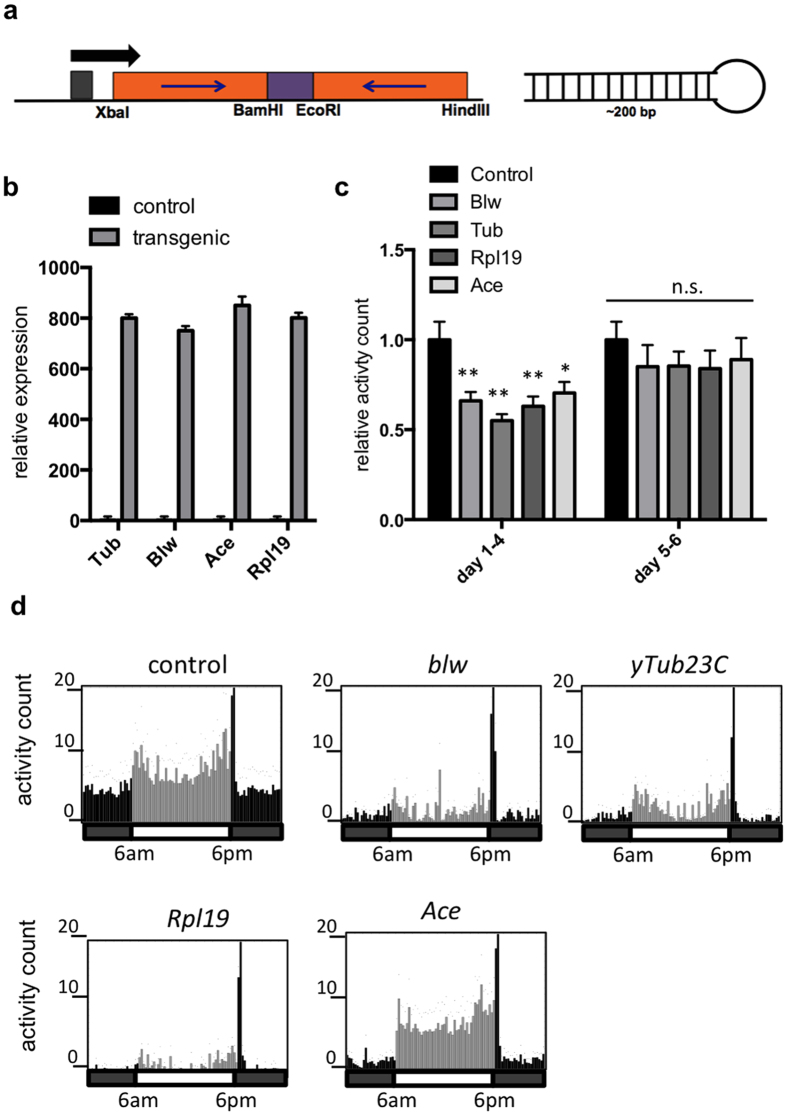
*D. suzukii* adults fed on yeast expressing dsRNA have decreased locomotor activity levels. (**a**) Map of transformation vector for expression of hairpin dsRNA in *S. cerevisiae*. ~200 bp of *D. suzukii* target gene sequence was inserted into p406TEF1 to form inverted repeats linked by 74 bp of intron sequence from the *white* gene. Black arrow indicates direction of transcription. (**b**) Expression of dsRNA in *S. cerevisiae.* Expression was quantified with RT-qPCR and was normalized against housekeeping gene *actin*. Data represent mean and s.e.m. of three technical replicates. (**c**) Locomotor activity levels in *D. suzukii* following a three-day treatment with yeast expressing dsRNA targeting *D. suzukii blw*, *yTub23C, Ace,* and *RpL19.* Male adult flies were fed *ad libitum* with a choice between artificial diet and live yeast for three days. Activity was recorded for 6 days after the feeding period using the DAMS. The first through the fourth days of recording were averaged together, and days 5 and 6 were averaged. Data represent mean and s.e.m. of 3 independent experiments, and a total of 96 flies were tested per treatment. Each experiment was normalized to the average of the control treatment. One-way ANOVA was performed (p = 0.0009) and significance relative to the control was determined by post hoc Dunnett corrected *t*-test (*indicates p < 0.05 and **indicates p < 0.01). (**d**) Activity levels plotted over the second day of recording following three days of feeding treatment. Each bar represents average activity counts in 15-minute increments. Black bars indicate lights off and grey bars represent lights on. Presence or absence of light as well as the timing of light transition are also indicated in the horizontal bar underneath each activity graph; light turns on at 6am and turns off at 6pm each day of recording. Each activity graph represents an average of 32 flies in one experiment. Representative activity graphs are shown here.

DNA plasmids were constructed to constitutively express dsRNA hairpins targeting *D. suzukii* critical genes *y-tubulin 23C (yTub23C)* (183 bp), *bellwether (blw)* (230 bp)*, Ribosomal protein L19 (RpL19)* (215 bp), and *acetylcholine esterase (Ace)* (221 bp). The rationale for selection of these target genes is stated in the methods section. Approximately 200 bp *D. suzukii* target gene sequence was inserted into a DNA plasmid to form inverted repeats joined by 74 bp of intron sequence from the *white* gene from *D. melanogaster* (Fig. 2a). This plasmid is self-replicating and does not integrate into the host yeast genome, so the host genome remains unchanged. Plasmids were transformed into *S. cerevisiae* strain INVSc1 using heat shock, and expression of dsRNA was verified using RT-qPCR (Fig. 2b). Empty P406TEF1 plasmid was transformed into *S. cerevisiae* strain INVSc1 to use as a control strain. As a control for dsRNA treatments, some studies use dsRNA targeting a gene from another species, such as GFP. We chose to use a yeast that contains empty plasmid and expresses no dsRNA as the control strain to avoid possible off target effects, such as suppression of critical genes that may have regions of sufficient homology to the control sequence to bring about an RNAi response^45^.

### *D. suzukii* adults fed on yeast expressing dsRNA have decreased locomotor activity levels

Initially, *D. suzukii* were continuously fed *ad libitum* for ten days with a choice between standard *Drosophila* artificial diet and live transformed yeast growing on solid agar. This feeding treatment with yeast expressing dsRNA targeting *yTub23C, blw*, *Ace*, or *Rpl19* resulted in 100% survival in all treated and control groups (data not shown). To examine possible sublethal effects on fitness following the feeding treatment, we measured locomotor activity as a proxy for general health. Adult *D. suzukii* were fed for three days on live yeast expressing dsRNA targeting *yTub23C*, *blw*, *Ace*, or *Rpl19*, and then were placed into glass tubes for activity monitoring. Adult *D. suzukii* had decreased locomotor activity in the 4 days following treatment (Fig. 2c) and the greatest decrease in activity was observed in the treatment targeting *yTub23C*. Although activity levels were reduced in flies treated with dsRNA, flies in all treatments maintained typical temporal patterns of activity through the day (i.e. anticipation of dawn and dusk, and peak of activity at dusk) (Fig. 2d). We found that this effect on locomotor activity level persisted over the first four days following the feeding period, but the activity levels of the treated flies were not significantly different from the control flies by the fifth or sixth day following the feeding period (Fig. 2c). This result indicates the treated flies were able to recover from the dsRNA treatment. Due to the fact that the yeast expressing *yTub23C* dsRNA produced the greatest reduction in activity levels, all subsequent molecular and physiological assays were only performed using *yTub23C*-targeting yeast.

### The effects of yeast feeding treatment on adult locomotor activity levels are species specific

**Figure 3:**
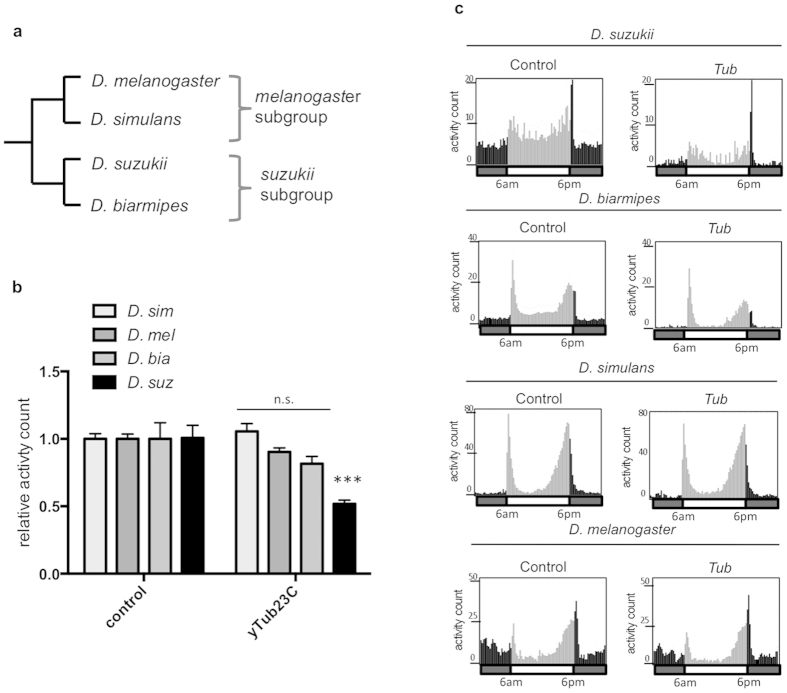
The effects of yeast feeding treatment on adult activity levels are species specific. (**a**) Cladogram of *melanogaster* and *suzukii* subgroups. Only the species tested were included in the cladogram. (**b**) Relative locomotor activity levels in *D. suzukii, D. melanogaster, D. simulans,* and *D. biarmipes* following a three-day treatment with yeast expressing dsRNA against *yTub23C*. Activity was recorded using the DAMS during the 4 days following the treatment. Data represent averages and s.e.m. of 3 independent experiments, and a total of 96 flies were tested per treatment. Each replicate was normalized to the average of the control treatment. One-way ANOVA was performed (p = 0.0001) and significance relative to the control was determined by post hoc Dunnett corrected *t*-test (***indicates p < 0.001). (**c**) Activity levels plotted over the second day of recording following three days of feeding treatment. Each bar represents average activity counts in 15-minute increments. Black bars indicate lights off and grey bars indicate lights on. Presence or absence of light as well as the timing of light transition are also indicated in the horizontal bar underneath each activity graph; light turns on at 6am and turns off at 6pm each day of recording. Each eduction graph represents an average of 32 flies in one experiment. Representative eduction graphs are shown here.

One benefit of using RNAi in pest management is that RNAi triggers can be designed to target pest species while minimizing the risk of effects on beneficial insects^46^. To demonstrate this, we tested a *D. suzukii* dsRNA fragment selected from the non-conserved *yTub23C* 3’ untranslated region (UTR). We fed transformed yeast expressing dsRNA targeting *D. suzukii yTub23C* to other Drosophilids and found that even closely related species were unaffected by the treatments (Fig. 3b). *D. biarmipes* is closely related to *D. suzukii*, while *D. melanogaster* and *D. simulans* are more distant relatives (Fig. 3a). *D. simulans, D. biarmipes,* and *D. melanogaster* were unaffected by treatment with yeast expressing dsRNA targeting *D. suzukii yTub23C*. All species maintained regular temporal patterns of activity throughout the day (Fig. 3c). Homologous gene fragments were identified for each species by BLAST sequence alignment^47^, and sequence identity scores were calculated (Supplementary Table S1). The *yTub23C* target gene fragment was designed to be divergent by targeting the 3’ UTR and is 90% to 74% conserved between *D. suzukii* and the other Drosophilids tested. We predicted the *yTub23C* fragment would be less effective on species other than *D. suzukii* due to reduced base pair matching. The lack of an effect on activity in species other than *D. suzukii* suggests that a high degree of perfect base pair matching is required for RNAi response and to reduce locomotor activity. These results imply the mechanism responsible for this reduction in locomotor activity level is target sequence dependent. An alternative explanation for the differences in activity level between species following treatment could be that the dosage, i.e. feeding rate, varies by species, since the feeding assay was conducted *ad libitum*.

### Ingestion of yeast biopesticide alters expression level of target genes with subgroup specificity

**Figure 4:**
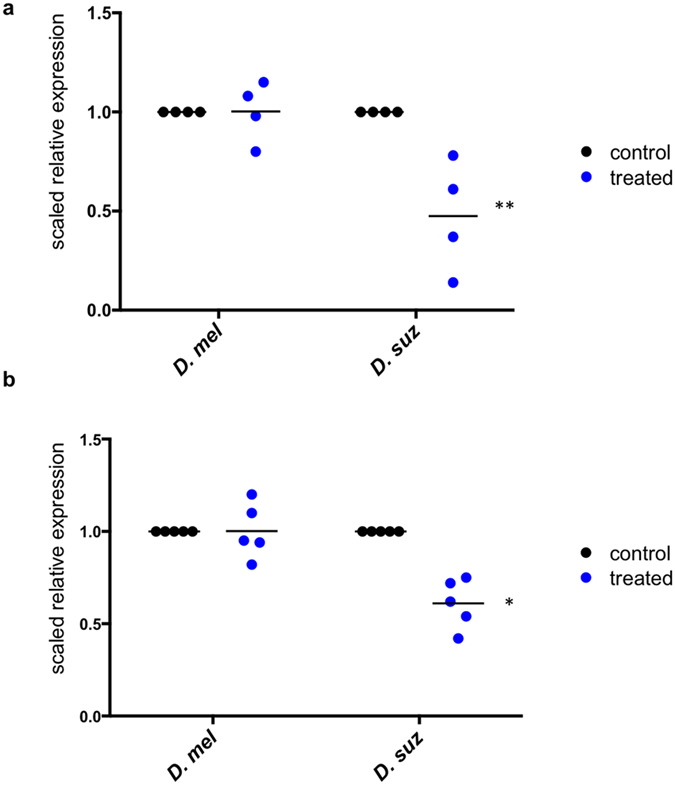
Altered target gene expression in midgut following yeast feeding treatment. Relative expression of target gene *y-tubulin 23C* in (**a**) adult midgut and (**b**) larval midgut following three days of treatment with yeast expressing dsRNA. Ten midguts were dissected and homogenized to obtain one RNA sample. Expression was quantified with RT-qPCR and was normalized against housekeeping gene *cbp20*. The expression level in the control is scaled to one and treated expression levels are plotted relative to the control. Each data point represents one biological replicate and the horizontal line indicates the mean. For (**a**) n = 4, and (**b**) n = 5. Significance relative to the control was determined by two-tailed *t*-test (*indicates p < 0.05, and **indicates p < 0.01).

RNAi is not systemic in *Drosophila*, meaning RNAi only occurs within cells that come into contact with dsRNA^48^. We examined the midgut tissues post-treatment to measure changes in target gene expression in adults and larvae (Fig. 4a,b). *D. suzukii* larvae fed on yeast expressing dsRNA targeting *yTub23C* for 72 hours had significantly reduced *yTub23C* mRNA levels in the midgut (Fig. 4b). Adult *D. suzukii* midgut gene expression following yeast treatment was similar to the results in larvae, i.e. *yTub23C* expression level was significantly decreased (Fig. 4a). We did not find significant changes in target gene expression in *D. melanogaster* larva or adult midguts following 72-hour feeding treatments with yeast expressing dsRNA targeting *D. suzukii yTub23C* (Fig. 4a,b). The lack of change in target gene expression in *D. melanogaster* was not surprising, since we also observed no changes in locomotor activity. Moreover, larvae were subjected to a no-choice feeding assay, so differences in gene expression between *D. suzukii* and *D. melanogaster* larvae are not expected to depend on dosage.

### Ingestion of yeast biopesticide reduces reproductive fitness

**Figure 5:**
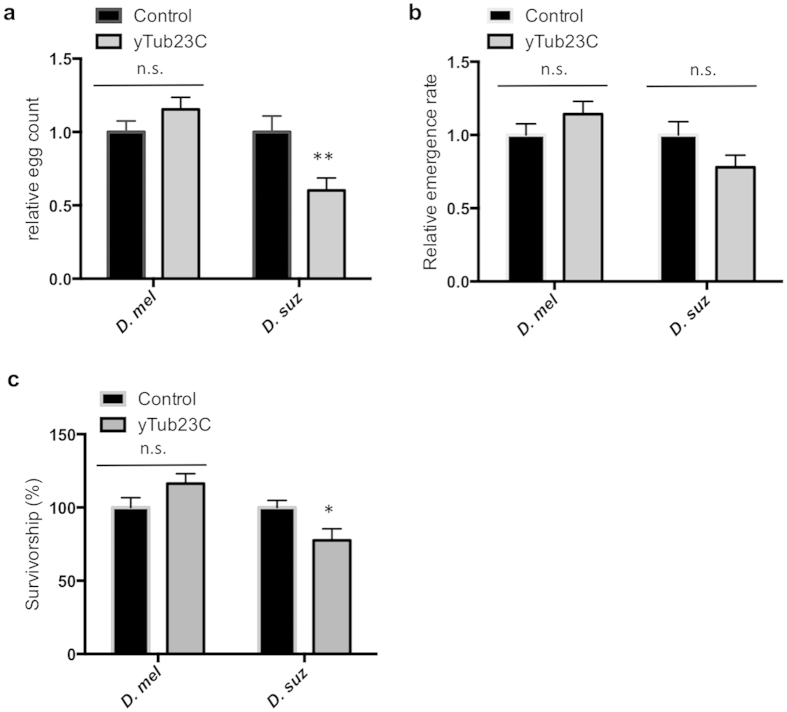
Yeast feeding treatment of adults and larvae decreases larval survivorship and adult reproductive fitness in a species specific manner. (**a**) Relative egg counts of *D. melanogaster* and *D. suzukii.* Virgin females and males were fed separately on yeast expressing dsRNA against *yTub23C* for three days. One male and one female were crossed and allowed to lay eggs for 48 hours (*D. melanogaster*) or 72 hours (*D. suzukii*). (**b**) Relative emergence rate of *D. melanogaster* and *D. suzukii* eggs when parents were fed as described in (**a**). The hatch rate was determined by dividing the number of adults by the number of eggs recorded in that vial. Data shown in (**a**,**b**) represent mean and s.e.m. of three independent experiments and a total of 75 females were tested per treatment. Each replicate was normalized to the mean of the control and significance relative to the control was determined by a two-tailed *t*-test (**indicates p < 0.01). (**c**) Survivorship of *D. melanogaster* and *D. suzukii* 2nd instar larvae following 24 hour treatment with live yeast expressing dsRNA against *yTub23C*. Larvae were fed on a mixture containing 50% artificial diet and 50% live-pelleted yeast by volume. Mortality was assessed at the time of eclosion. Data shown in (**c**) represent mean and s.e.m. of six independent experiments and a total of 300 larvae were tested per treatment. Each replicate was normalized to the mean of the control and significance relative to the control was determined by a two-tailed *t*-test (*indicates p < 0.05).

Sublethal effects of pesticides, such as behavioral changes, general health decline, and decreased reproductive fitness, can lead to reduced population size within a region, minimizing crop losses^49^. To test if the yeast treatment affects reproductive fitness, we counted the number of eggs deposited per female post-treatment and measured the survival rate to adulthood of the offspring. We found that yeast expressing dsRNA that targets *D. suzukii yTub23C* caused significant decrease in the number of eggs laid by *D. suzukii*, but not by *D. melanogaster* (Fig. 5a). One possible explanation of this result is that induction of RNAi targeting *yTub23C* reduces overall fitness, so females are unable to devote resources towards producing eggs. An alternative explanation is that because treated flies are less active, the first mating is delayed compared to the control, resulting in fewer eggs at the end of the test period.

We propose that the reduction in egg-laying by *D. suzukii* females following the transformed yeast feeding treatment is due to RNAi mediated disruption of *yTub23C* expression level. This altered expression level may cause cell death or dysfunction in the midgut, which could lead to decreased nutrition and general health decline. It is possible that disruption of midgut function can negatively impact nutritional input and reproduction, explaining the observed reduction in fecundity. We also hypothesized that decreased parental health following the yeast feeding treatment could lead to reduced viability of eggs. To test this hypothesis, we recorded survival rate to adulthood of eggs when the parents were treated with transformed yeast. The survival rates of the offspring of treated adults as measured by adult emergence rate were unaffected in both species (Fig. 5b).

### Ingestion of yeast biopesticide decreases larval survivorship

Ingestion of yeast expressing dsRNA targeting *D. suzukii yTub23C* during the larval stages decreased survival to adulthood in *D. suzukii*, but not in *D. melanogaster* (Fig. 5c). Relative to the control treatment, which has a survival rate of 72%, the *yTub23C* treatment significantly reduced *D. suzukii* larval survival by 23% (Supplementary Table S2). The survival rate of *D. melanogaster* larvae subjected to the *yTub23C* treatment was not significantly different (Fig. 5c). Adult *D. suzukii* fed on yeast expressing dsRNA targeting *yTub23C, Ace, RpL19, blw*, and the control yeast had 100% survival over a ten-day feeding period (data not shown). Larvae consume a greater amount of food per day compared to adults. Since the larvae were fed in a no-choice assay, and the adults were fed with a choice between live transformed yeast and artificial diet, we reason that larvae likely received higher dosage of yeast. Differences in dosage could explain why the yeast feeding treatment induces mortality in larvae, but not in adults. Alternatively, we speculate larval stages could be more susceptible to RNAi than adults due to differences in the absorptive properties of the midgut or robustness of the RNAi pathway.

## Discussion

Here we show that genetically modified yeast can be used to deliver dsRNA to an insect pest, causing decreased fitness through RNAi. Ingestion of this modified yeast by adult *D. suzukii* results in decreased locomotor activity and reduced egg-laying. Larvae exhibit reduced survival to adulthood when fed on yeast expressing dsRNA, and adults and larvae have decreased target gene expression following yeast feeding treatment. We find the effects of the yeast feeding treatment on target gene expression, survival, and reproductive fitness are subgroup specific, i.e. *D. suzukii* is negatively impacted while *D. melanogaster* is not. It should be noted that we did not perform experiments to confirm that dsRNA produced by the yeast was present within the insect cells following ingestion of the yeast. We found there was no feasible assay to separate the contents of the gut from the midgut cells and accurately detect exogenous transcripts coding for *D. suzukii* genes within *D. suzukii* cells. Additionally, it should be noted that the control yeast strain contains only empty plasmid and does not produce any dsRNA. Therefore, differences between the control and dsRNA treated samples may not be exclusively due to the effects of RNAi alone. It is possible that the dsRNA decreases fitness by inducing a sequence-independent response such as activation of the immune system, a phenomenon that has been observed in mammalian cells^50^. However, since species with divergent target gene sequences, such as *D. simulans*, were unaffected by the yeast feeding treatment, we find it unlikely that sequence-independent effects were responsible for the observed decreases in fitness. Changes in target gene expression following yeast-feeding treatment were observed in *D. suzukii* but not in *D. melanogaster* (Fig. 4). *D. biarmipes, D. melanogaster,* and *D. simulans* exhibited no changes in locomotor activity (Fig. 3b). Together, these results suggest that sequence-dependent target gene knockdown via RNAi was the primary cause of decreased fitness observed in *D. suzukii*.

This yeast biopesticide could be improved as a delivery vehicle by additional modifications to the yeast genome. A potential improvement to the current design could be to create modifications such that dsRNA is excreted from the yeast cells in membrane-bound vesicles. In the current design, dsRNA is expressed from circular DNA plasmids and remains within the cell. We hypothesize that dsRNA is absorbed into insect cells only when yeast cells are lysed within the insect midgut. It has been previously demonstrated that lipid encapsulated siRNA is more stable and more readily absorbed than naked siRNA^51^. Therefore, we predict continuous excretion of dsRNA in membrane bound-vesicles from intact yeast could increase the dosage and absorption of dsRNA into insect midgut cells.

The biosafety of this yeast biopesticide could be increased by adding a built-in mechanism of containment. A common concern with genetically modified organisms is their containment and potential negative impact on the environment. Yeast can be carried from one location to another by *Drosophila*, so it seems likely that genetically modified yeast could be spread beyond the boundaries of agriculture. Our design could be improved simply by placing the dsRNA under a promoter that requires the presence of an artificial molecule in order to prevent expression of dsRNA in the yeast where it is not wanted^52^. Recently, bacteria known as synthetic auxotrophs were engineered to only be viable in the presence of the synthetic molecule benzothiazole^53^, which prevents genetically modified bacteria from escaping and increases biosafety. However, benzothiazole is toxic and may pose additional problems for human safety. Another genetic modification technique has been used to create bacterial strains that require synthetic amino acids for growth^54^, which may be more suitable for environmental use. This technology could be applied to yeast biopesticides to prevent unwanted escapes.

We predict that this yeast biopesticide could be successfully implemented in the field to combat *D. suzukii*. The emergence of insecticide resistance and increasing popularity of organic food products may open the door to non-chemical means of pest control in both organic and conventional farming. A method for administering this yeast biopesticide in the field has already been presented, since yeast baited traps have previously been used in berry crops to lure *D. suzukii*^40^. Additionally, there is precedence for the agricultural use of genetically modified fungi, as the USDA has issued 26 permits for environmental release of genetically modified fungi since 1995^55^. A major limitation of the current design is that mortality is not induced in adults. However, we found that this yeast biopesticide significantly decreases egg-laying and therefore could decrease the population size of the next generation. This yeast biopesticide could be used in complement with chemical pesticides by targeting detoxification genes rather than critical genes, a strategy that has proven effective for increasing susceptibility of mosquito larvae to insecticides^24^. By altering expression of detoxification genes, *D. suzukii* could be made more susceptible to chemical pesticides. As different types of insecticide resistance emerge, the genes targeted by the yeast could easily and quickly be changed to compensate. Further screening and validation is required to identify target genes that will have the greatest efficacy and produce high level of lethality when knocked down in the field. Targeting multiple genes simultaneously may also increase toxicity of this biopesticide, although a recent study conducted in *Tribolium castaneum* found that the effects of targeting multiple genes are not synergistic^56^.

Another characteristic of yeast that makes it an interesting candidate for a biopesticide is that unlike chemical pesticides, it can be easily grown with commonplace starting materials. Currently, live *S. cerevisiae*, commonly known as brewer’s yeast, can be purchased in packets from any grocery store. The yeast in these packets is dry and does not require refrigeration. We envision that in the future, a yeast biopesticide could be similarly packaged and distributed to farmers in developing countries, where it could be cultured at the site of use.

This yeast biopesticide design could be adapted to deliver dsRNA to other insect pests that associate with yeast. Many interactions have been observed between wild yeasts and insect species within Hymenoptera, Coleoptera, and Diptera^57^. In fact, this biopesticide design may have greater use in managing an insect pest that both consumes yeast and has systemic RNAi.

A limitation of all RNAi-based technology is potential off-target effects. Only partial complementarity between the guide strand and the endogenous mRNA is required to prevent translation, so silencing of related transcripts in humans and other non-target species is a concern. Non-target arthropod species that have systemic RNAi mechanisms may especially be at risk, and many of these species do not have genomic data available to facilitate the design of pest-specific dsRNAs^46^. In addition to sequence-dependent off-target effects, introduction of dsRNA into mammalian cells can have sequence-independent effects on the immune system^58^. For example, dsRNA can activate an innate immune response in mammals, resulting in increased levels of inflammatory cytokines such as tumor necrosis factor and interferons^50^. In this pathway, dsRNA binds and activates protein kinase R, which phosphorylates and deactivates eukaryotic initiation factor eIF2, thereby inhibiting translation in cells that contain dsRNA^59^. Since these effects on the immune system are not dependent on the sequence of the dsRNA, even careful selection of target sequences cannot mitigate these off-target effects. Pesticides containing dsRNA will need further testing to determine safety for non-target arthropods and for human consumption.

## Methods

### Animal Models

For all insect bioassays, the researcher was blinded from the identity of the treatment during the course of the experiment. Insects were assigned to treatment groups by pooling same-aged individuals from several rearing bottles and randomly redistributing into groups. A different generation of flies was used in each experimental replicate.

### Statistics

Statistical analysis was performed with GraphPad Prism version 6.0f. The student’s t-test was used in assays that compared two groups. For assays with more than two groups, one way or two-way ANOVA was performed using 95% confidence intervals to determine significance. Significance relative to control treatments was determined by post hoc t-test with Dunnett correction for multiple comparisons. To allow for comparisons between species, each data point or replicate is normalized to the mean of the same-species control group (Figs 3b and 5)^60^.

### Fly strains and rearing

All *Drosophila* species and strains tested in our studies as well as their collection sites are listed in Supplementary Table S3. All lines were maintained in Fisherbrand square, polyethylene, 6 oz. stock bottles (Fisher Scientific, Pittsburgh, PA) containing 50 ml of Bloomington stock center *Drosophila* food recipe. Colonies were kept between 22 °C–25 °C in a cabinet incubation chamber (Percival Scientific, Inc., Perry, IA) with a 12:12 h light:dark cycle.

### Target gene selection for double stranded RNA (dsRNA) knockdown

Target genes were selected based on four main criteria: gene essentiality, midgut expression level, degree of divergence from related species sequences (Supplementary Table S1), and target effectiveness demonstrated in previous studies. We assumed that genes with essential functions in *D. melanogaster* would also have essential functions in *D. suzukii*. Genes with lethal null phenotypes recorded in Flybase.org were considered essential. We hypothesized that the dsRNA produced by the yeast would be absorbed by the midgut tissues in the fly, so we selected target genes that are expressed in the adult and larval midgut. Midgut expression was determined using the FlyAtlas database of microarray data^61^. Conservation of target sequences between species was also considered when selecting gene fragments. The *D. suzukii y-tubulin 23C* (*yTub23C*) target sequence was designed to be highly divergent from members of the *melanogaster* subgroup by targeting the 3’ untranslated region (UTR). The *bellwether* (*blw*), *Ribosomal protein L19* (*RpL19*), and *acetylcholine esterase* (*Ace*) target sequences were chosen from coding regions and are more conserved across species. Alignments of target gene fragment sequences (Supplementary Fig. S1) for *D. suzukii, D. biarmipes, D. melanogaster,* and *D. simulans* and identity scores (Supplementary Table S1) were generated with ClustalW2^62^. Whyard *et al.*^20^ demonstrated that *tubulin* is an effective RNAi target in *Drosophila*, and dsRNA targeting *blw* and *RpL19* caused increased cell death in *D. melanogaster* tissue culture experiments^63^, so we selected homologous target gene fragments from *D. suzukii* sequences. Chemical pesticides commonly disrupt neurotransmission by inhibiting the function of acetylcholine esterase, so we chose to target this gene as well. Target gene fragment sequences are listed in Supplementary Fig. 2. Spotted Wing Flybase accession numbers were used as identifiers and sequences can be retrieved from Spotted Wing Flybase (http://spottedwingflybase.oregonstate.edu/).

### Construction of vectors for *in vitro* synthesis of dsRNA

1 μg of *D. suzukii* total RNA was used to synthesize cDNA using ThermoScript RT-PCR System (Life Technologies, Grand Island, NY) according to the manufacturer’s protocol. Target gene fragments *y-tubulin 23C* and *Vacuolar H*+*-ATPase 26* *kD* subunit were PCR amplified from *D. suzukii* total cDNA using AccuPrime Taq DNA Polymerase (Life Technologies, Grand Island, NY) following manufacturer’s specifications and ligated into pCR2.1 using the TA Cloning Kit (Life Technologies, Grand Island, NY). The primer sequences used for molecular cloning are listed in Supplementary Table S4. The T7 promoter sequence (5′- TAATACGACTCACTATAGG-3′) was added to the 5’ end of the forward and reverse primer sequences.

### *In vitro* RNA synthesis

Target gene fragments *y-tubulin 23C* and *vacuolar H*+*-ATPase 26* *kD* subunit were PCR amplified from pCR2.1 vectors containing the target sequences and convergent T7 promoters. PCR products were purified with the QiaQuick PCR Purification Kit (Qiagen, Valencia, CA). Purified PCR product was used as template for *in vitro* transcription using the MEGAscript T7 Transcription Kit (Life Technologies, Grand Island, NY) following manufacturer’s instructions. RNA concentrations were quantified with a NanoDrop 2000 Spectrophotometer (Fisher Scientific, Pittsburgh, PA), and quality was assessed by electrophoresis on 1% agarose gel.

### Construction of plasmid vectors for expression of dsRNA in yeast

Yeast expression vectors containing inverted repeats of ~200 bp *D. suzukii* target gene sequence joined by a 74 bp intron sequence from the *D. melanogaster white* gene were constructed in two cloning steps. The intron and the target sequence were inserted in the forward orientation in the first step, and the target sequence was inserted in the reversed orientation in the second step. Target gene fragments *y-tubulin 23C, bellwether, acetylcholine esterase,* and *ribosomal protein L19* were PCR amplified from *D. suzukii* total cDNA using AccuPrime Taq DNA Polymerase (Life Technologies, Grand Island, NY) following manufacturer’s specifications. A BamHI restriction site was added to the 5’ end of the forward primer sequence, and a EcoRI restriction site was added to the 5’ end of the reverse primer sequence (see Supplementary Table S4 for primer sequences). The second intron of the *white* gene was PCR amplified from *D. melanogaster* total cDNA using primers with a BamHI restriction site added to the 5’ end of the forward and a EcoRI site added to 5’ end of the reverse primer. PCR products were purified with the QiaQuick PCR Purification Kit (Qiagen, Valencia, CA) and target sequences were digested with restriction enzymes BamHI and Xba1, and the *white* intron was digested with BamHI and EcoRI (New England Biolabs, Ipswitch, MA). The *S. cerevisiae* expression vector p406TEF1 (Addgene, Cambridge, MA) was digested with Xba1 and EcoRI. Digested PCR products and vector DNA were purified with gel electrophoresis and the QiaQuick Gel extraction kit (Qiagen, Valencia, CA) following manufacturer’s instructions. The vector, target sequence, and intron were ligated together with T4 DNA ligase (New England Biolabs, Ipswitch, MA). Ligation products were transformed into *E. coli* and plasmid DNA was extracted from the clones. Sequencing was performed to confirm presence of the forward target sequence and the intron. Target gene fragments were amplified from the confirmed DNA clones using a reverse primer with EcoRI on the 5’ end and a forward primer with HindIII on the 5’ end. Confirmed DNA clones were restriction digested with EcoRI and HindIII. Digested PCR products and DNA vectors were gel purified and ligated together with T4 DNA ligase. The complementary sequences in the completed vectors created secondary structure that prevented Sanger sequencing, so completed clones were restriction digested with Xba1 and EcoRI, or BamHI and HindIII, and gel purified digestion products were used for sequencing for confirmation of successful expression vector construction.

### Transformation of plasmids into *S. cerevisiae*

DNA vector p406TEF1 containing inverted repeats of *D. suzukii* target gene sequence were transformed into *S. cerevisiae* strain INVSc1 (Life Technologies, Grand Island, NY) using the Frozen EZ Yeast Transformation kit II (Zymo, Irvine, CA) following manufacturer’s instructions. Transformants were selected on minimal media without uracil and confirmed by colony PCR. PCR reactions were performed as described above with a forward primer located upstream of the multiple cloning site and the reverse primer located within the *white* intron.

### Isolation of RNA from yeast

1.5 mL liquid yeast culture was pelleted at 12,000 rpm for 1 minute. 300 μl of TRI-reagent (Sigma Aldrich, St Louis, MO) was added to the pellet and homogenized by vortexing with 100 μl acid-washed 425–600 μm glass beads (Sigma Aldrich, St Louis, MO). One-fifth volume of 100% chloroform was added to each sample and incubated at room temperature for 10 minutes. Samples were centrifuged at 13,000 rpm for 15 minutes at 4 °C. The upper aqueous layer was collected and nucleic acid was precipitated by adding an equal volume of 100% isopropanol and incubated at room temperature for 10 minutes. Samples were centrifuged at 13,000 rpm for 15 minutes at 4 °C, and pellets were washed once with two volumes of 70% ethanol. Pellets were resuspended in 20 μl 1× Turbo DNase buffer (Life Technologies, Grand Island, NY) and treated with 1 μl Turbo DNase following manufacturer’s instruction. RNA concentrations were quantified with a NanoDrop 2000 Spectrophotometer (Fisher Scientific, Pittsburgh, PA).

### Treatment of larvae with *in vitro* transcribed dsRNA

2nd instar larvae were separated from fly food and rinsed with water. 100 μl of a solution containing 1 mg/ml *in vitro* transcribed dsRNA, 3% Lipofectamine 2000 (Life Technologies, Grand Island, NY), and Schneider’s *Drosophila* medium (Life Technologies, Grand Island, NY) was pipetted onto a plastic dish forming a droplet. The control solution contained Lipofectamine and Schneider’s *Drosophila* medium only. 10 larvae were gently added to each droplet and soaked for 1 hour. Larvae were returned to a small dish of fly food and mortality was assessed 24 hours later and at the time of eclosion. Larvae were collected for RNA isolation at the same time-point when mortality was assessed. Three independent experiments were performed using larvae from three separate generations, and 50 to 60 larvae were tested per treatment in each replicate experiment.

### Transformed yeast feeding assay for adults and larvae

Yeast colonies expressing dsRNA were incubated in 4 ml minimal media without uracil at 30 °C and 225 rpm for 24 hours. 4 ml cultures were used to inoculate 300 ml cultures and incubated at 30 °C for 24 hours. Liquid culture was pelleted at 5,000 rpm for 15 minutes. To feed larvae, pelleted yeast was mixed with standard Bloomington *Drosophila* fly food medium at a ratio of 1:1 by weight and fed to larvae in 12- well tissue culture plates. 1 mL of food and yeast mixture and twenty larvae were added to each well. After yeast feeding period, larvae were transferred to a vial containing standard Bloomington *Drosophila* medium to grow to adulthood, or were collected for RNA isolation. Larvae were fed for 24 hours for survival assays, and for 24 hours or 72 hours for gene expression assays. To quantify survival, larvae were fed on yeast for 24 hours and transferred to a vial in groups of 20. A moistened lab wipe was added to each vial to increase the humidity and provide a surface for pupation. The number of adults that emerged from each vial was used as a measure of survival.

To feed adults, pelleted yeast was spread thinly on a wooden tongue depressor coated in YPD agar (Sigma Aldrich, St Louis, MO). The yeast-covered tongue depressor was imbedded upright in a vial containing standard Bloomington *Drosophila* medium and 30 adult flies were added to each vial.

To confirm the adults and larvae ingest the yeast, red food coloring was added to the liquid yeast culture (McCormick), and midguts were dissected and inspected visually under a light microscope. The control diet was standard Bloomington *Drosophila* medium without food coloring. Five adult midguts or larval midguts were dissected for each treatment. This experiment was performed twice.

### Locomotor activity monitoring

Male adult flies were used for activity assays because females present technical difficulties, e.g. larvae and eggs interfere with infrared monitoring and gravid females exhibit different activity profiles than non-gravid females. Flies were loaded individually into glass tubes for activity monitoring using *Drosophila* Activity Monitoring System (DAMS) (Trikinetics, Waltham, MA) following three days of yeast feeding treatment. The glass tubes were filled at one end with media containing 5% agar and 2% sucrose by weight. Percival incubators were set to 25 °C with a photoperiod of 12 hours of light and 12 hours of dark. Fly activity monitoring and data analysis were performed using DAMS and FaasX respectively^64^. Briefly, the DAMS consists of a infrared beam positioned to intersect the glass tube housing a single fly. When the fly travels from one end of the tube to the other, the infrared beam is broken and the DAMS records one activity count.

### Assessment of fecundity and viability of offspring

Standard Bloomington *Drosophila* medium was dyed green with food coloring (McCormick) to provide visual contrast to eggs. 1 ml of hot media was pipetted onto a small plastic spoon and allowed to cool. Once solidified, media was painted with a thin layer of wild type *S. cerevisiae* grown in liquid culture to encourage oviposition. Spoons were placed inside empty fly vials (Genesee Scientific, San Diego, CA). Adult males and virgin females were fed on transformed yeast expressing dsRNA for three days. Following the feeding period, one male and one female were added to the vial containing the spoon. After the egg-laying period, eggs were counted visually under a stereomicroscope. The spoon was transferred to a vial containing *Drosophila* medium and eggs were allowed to grow to adulthood. Survival rates were calculated by dividing the number of emerged adults by the number of eggs recorded in each vial.

### Isolation of RNA from adult and larval midgut

Following yeast feeding treatment, live adults were anesthetized with carbon dioxide and live larvae were separated from yeast mixture. Adults and larvae were transferred to a microcentrifuge tube containing TRI-reagent (Sigma Aldrich, St Louis, MO) and kept on ice. Dissection of the midgut was performed immediately on ice in chilled RNAlater (Life Technologies, Grand Island, NY). Midguts were washed 2× in chilled nuclease-free water and 300 μl TRI-reagent was added to the microcentrifuge tube. Midguts were homogenized by grinding using a motorized pestle and RNA isolation was performed as described above in the section on “yeast RNA isolation”.

### Quantitative real-time PCR (qRT-PCR)

1 μg of total RNA was used to synthesize cDNA using ThermoScript RT-PCR System (Life Technologies, Grand Island, NY) according to the manufacturer’s protocol. Dilutions (1:10) of cDNA samples were used in qRT-PCR reactions. Gene-specific primers were designed based on sequence analysis using the *D. suzukii* genome scaffold and optimized at an annealing temperature of 63.3 °C. The primer efficiencies for *D. melanogaster yTub23C* and *D. suzukii yTub23C* were 99.1% and 100.2% respectively. Melt curve and BLAST analysis were used as criteria to determine primer specificity. The qRT- PCR assays were performed using SsoAdvanced SYBR Green Supermix (Bio-rad, Hercules, CA) in a CFX96 Touch Real-Time PCR Detection thermal cycler (Bio-Rad, Hercules, CA). Cycling conditions were 95 °C for 30 seconds, 40 cycles of 95 °C for 5 seconds, followed by an annealing/extension phase at 63.3 °C or 55 °C for 30 seconds. The reaction was concluded with a melt curve analysis going from 65 °C to 95 °C in 0.5 °C increments at five seconds per step. Three technical replicates were performed for each data point of each biological replicate, and at least 4 biological replicates were performed for analysis of each gene. Data were analyzed using the standard ∆∆Ct method and target gene mRNA expression levels were normalized to the reference gene *Cbp20* mRNA levels^65^,^66^. Finally, average relative expression values for treated samples were divided by the average control values to represent the fold change of target gene expression in the treated sample^65^.

## Additional Information

**How to cite this article**: Murphy, K. A. *et al.* Ingestion of genetically modified yeast symbiont reduces fitness of an insect pest via RNA interference. *Sci. Rep.* **6**, 22587; doi: 10.1038/srep22587 (2016).

## Supplementary Material

Supplementary Information
