# Supplementary material for: Ingestion of genetically modified yeast symbiont reduces fitness of an insect pest via RNA interference
Source: Sci Rep. 2016 Mar 2;6:22587. doi: 10.1038/srep22587 (PMC4773866; doi:10.1038/srep22587)
Supplement: Supplementary Information [file srep22587-s1.pdf]

Supplementary Information

Title: Ingestion of genetically modified yeast symbiont reduces fitness of an insect pest via RNA interference

Authors: Katherine A. Murphy, Christine A. Tabuloc, Kevin R. Cervantes, Joanna C. Chiu

Supplementary Table S1: Sequence conservation of target gene fragments

Symbol	Species	Identity	Longest match	NCBI accession
blw	*D. biarmipes*	99%	130 bp	KB462602
blw	*D. melanogaster*	96%	42 bp	NM_166554
blw	*D. simulans*	95%	50 bp	CM002911 JPYS01000000
yTub23C	*D. biarmipes*	90%	47 bp	KB462641 AFFD02000000
yTub23C	*D. melanogaster*	76%	15 bp	NM_057456
yTub23C	*D. simulans*	74%	16 bp	XM_002077929 CM002910
RpL19	*D. biarmipes*	98%	68 bp	KB462460.1
RpL19	*D. melanogaster*	94%	38 bp	NM_206219.2
RpL19	*D. simulans*	94%	38 bp	XM_002083026
Ace	*D. biarmipes*	94%	38 bp	KB462068.1
Ace	*D. melanogaster*	93%	64 bp	NM_001275601.1
Ace	*D. simulans*	94%	74 bp	XM_002103562

Supplementary Table S2: Larval survival when fed yeast expressing dsRNA targeting *yTub23C*

Species	Treatment	Sample size	Replicates	Total number of larvae	Average survival %	Normalized survival	Normalized s.e.m.	P value
D. suz	Control	20	15	300	72.9	1	0.048	
D. suz	*yTub23C*	20	15	300	63.2	0.77	0.078	0.024
D. mel	Control	20	15	300	58	1	0.067	
D. mel	*yTub23C*	20	15	300	67.2	1.16	0.068	0.103

Supplementary Table S3: *Drosophila* strains used in this study

Species	Strain	Location of collection	Collector/Source
D. suzukii	Mixed Watsonville population	Watsonville, CA	Zalom Lab, UCD
D. biarmipes	genome strain, 361.0-isol e-11	Ari Ksatr, Cambodia	DSSC at UCSD
D. melanogaster	Oregon R	Roseburg, Oregon	DSSC at UCSD
D. simulans	W501	Genome Strain	DSSC at UCSD

Supplementary Table S4: Primers for molecular cloning and Quantitative PCR

Purpose	Primer Name	Species	C°	Sequence
qPCR	CBP20 F	*D. suzukii*	63.3	AGGAGCAGATCCACGAGCTC
qPCR	CBP20 R	*D. suzukii*	63.3	ACTCCACGAAGCAGAAGCC
qPCR	Tubulin F	*D. suzukii*	63.3	AATGTTTACCTATCCAAGCATG
qPCR	Tubulin R	*D. suzukii*	63.3	TCGAGTGGCAGAGTATGAATC
qPCR	Actin F	*S. cerevisiae*	63.3	ATTATATGTTTAGAGGTTGCTGCTTTGG
qPCR	Actin R	*S. cerivisiae*	63.3	CAATTCGTTGTAGAAGGTATGATGCC
qPCR	Blw dsRNA F	*dsRNA*detection[1]	63.3	AGGCTGTGGCCTACCGTCA
qPCR	Blw dsRNA R	*dsRNA* detection	63.3	AGGGAGCCACCTCCCATG
qPCR	Tub dsRNA F	*dsRNA* detection	63.3	ACGGACGGTCAGGTACTAGC
qPCR	Tub dsRNA R	*dsRNA* detection	63.3	TGCGAGTCTTATAAACAATGTGCT
qPCR	Ace dsRNA F	*dsRNA* detection	63.3	TGAACAACATTTTCGGCAAG
qPCR	Ace dsRNA R	*dsRNA* detection	63.3	TGGGCATACTCGTTGGTG
qPCR	Rpl19 dsRNA F	*dsRNA* detection	63.3	TACCGTGTGCGCAAGAAC
qPCR	Rpl19 dsRNA R	*dsRNA* detection	63.3	TGTCGATCTTCTTGCTGTCG
qPCR	CBP20 F	*D. melanogaster*	55	GTCTGATTCGTGTGGACTGG
qPCR	CBP20 R	*D. melanogaster*	55	CAACAGTTTGCCATAACCCC
qPCR	Tubulin F	*D. melanogaster*	55	TTTCCCAATCAGGACGAGATC
qPCR	Tubulin R	*D. melanogaster*	55	TGTTGATCTGCGAAAAGCTG
cloning	Ace(Xba1)F	*D. suzukii*		GTGT TCTAGA TGCCACGGGACAAATATCTC
cloning	Ace(HindIII)F	*D. suzukii*		GTGT AAGCTT TGCCACGGGACAAATATCTC
cloning	Ace(BamHI)R	*D. suzukii*		GTGT GGATCC GTAGTAGTGCACGGAGGCGC
cloning	Ace(EcoRI)R	*D. suzukii*		GTGT GAATTC GTAGTAGTGCACGGAGGCGC
cloning	Blw(Xba1)F	*D. suzukii*		GTGT TCTAGA ACGCCCTGATCATCTACGAC
cloning	Blw(HindIII)F	*D. suzukii*		GTGT AAGCTT ACGCCCTGATCATCTACGAC
cloning	Blw(BamHI)R	*D. suzukii*		GTGT GGATCC TGGAATGTAGGCGGACACATC
cloning	Blw(EcoRI)R	*D. suzukii*		GTGT GAATTC TGGAATGTAGGCGGACACATC
cloning	Tub(Xba1)F	*D. suzukii*		GTGTTCTAGAACGCTAAGTCGGAGGAC
cloning	Tub(HindIII)F	*D. suzukii*		GTGTAAGCTTACGCTAAGTCGGAGGAC
cloning	Tub(BamHI)R	*D. suzukii*		GTGT GGATCC ACAGTATTAACATACATGCG
cloning	Tub(EcoRI)R	*D. suzukii*		GTGTGAATTCACAGTATTAACATACATGCG
cloning	Rpl19(Xba1)F	*D. suzukii*		GTGT TCTAGA GGTCTGATCATCAAGAAGCCC
cloning	Rpl19(HindIII)F	*D. suzukii*		GTGT AAGCTT GGTCTGATCATCAAGAAGCCC
cloning	Rpl19(BamHI)R	*D. suzukii*		GTGT GGATCC AGGTGCCTGTCGATCTTCTTG
cloning	Rpl19(EcoRI)R	*D. suzukii*		GTGT GAATTC AGGTGCCTGTCGATCTTCTTG
cloning	White intron F	*D. melanogaster*		GTGTGGATCCGTGAGTTTCTATTCGCAG
cloning	White intron R	*D. melanogaster*		GTGTGAATTCCTGAGTTTCAAAATTGGTA
cloning	Tub(T7)F	*D. suzukii*		GAATAATACGACTCACTATAGGGAGAACGCTAAGTCGGAGGAC[2]
cloning	Tub(T7)R	*D. suzukii*		GAATAATACGACTCACTATAGGGAGAACAGTATTAACATACATGCG
cloning	vATPase(T7)F	*D. suzukii*		GAATAATACGACTCACTATAGGGAGATGGAGCAGTACAAGGCC
cloning	vATPase(T7)R	*D. suzukii*		GAATAATACGACTCACTATAGGGAGAAACAGGGCGTTACGAATC

[1]Primers listed with the term "dsRNA detection" were used to detect expression of dsRNA coding for *D. suzukii* gene sequences in *S. cerevisiae*.
[2] The underlined portion of the primer indicates the T7 promoter sequence.

Supplementary Figure S1: Alignments of *yTub23C, blw, Rpl19, and Ace D. suzukii* target gene fragments to *D. biarmipes, D. melanogaster, and D. simulans*. (* represents conserved nucleotides)

Alignments for *yTub23C*

```
Dsuz_tub        ACGCTAAGTCGGAGGACGGACGGTCAGGTACTAGCGGCGGTGTCTAGTTTGC--TCTTGC
Dbia_tub        ACACCAAGTCGGAGGACGGACGGTCTGCGACCAGCGGCAGTGTCTAGTTTACCATCGTGC
                ** * ******************** *  ** ****** *********** *  ** ***

Dsuz_tub        CATCAACAATGCGTGCCATGCCTTTTCTCGAATGTATTTTACAATTTCTGAAGACGTCGG
Dbia_tub        CATCAGCAATGCGTGCCACGCATTTTCTCGAATGTATTTTTCAATTTCTCAAGACGTCGG
                ***** ************ ** ****************** ******** **********

Dsuz_tub        GATTGGAAATCCCAAAGTATTAATAAGCACATTGTTTATAAGACTCGCATGTATGTTAAT
Dbia_tub        GATTGGAAATCCCAAAGTATTAATAAGCACATTGTTTGTAAGACTCGCATCTATGTTAAT
                ************************************* ************ *********

Dsuz_tub        ACTGT
Dbia_tub        GCTGT
                 ****
```

```
Dsuz_tub        ACGCTAAGTCGGAGGACGGACGGTCAGGTACTAGCGGCGGTGTCTAGTTTGCTCTTGCCA
Dmel_tub        ACTCCAAGTCGGAGGATAGTCGATCTGTGACCAGCGCCGGTTCCTAGTTCGCTTGTGCCA
                ** * ***********  * ** ** *  ** **** ****  ****** ***  *****

Dsuz_tub        TCAACAATGCGTGCCATGCCTTTTCTCGAATGTATTTTACAAT--------TTCTGAAGA
Dmel_tub        GAAGAAATGCGTGCCATGCATCTTAACCAATGTATTTTAAAATGTAATATTCTCCAATGA
                  *  ************** * **  * *********** ***         **  * **

Dsuz_tub        CGTCGGGATTGGAAATCCCAAAGTATTA---ATAAGCACATTGTTTATAAGACTCGCATG
Dmel_tub        CGATGGGATTCGAAATCCTTAAGTAGTAGTGATAAGACGATTGTTGGTATGACTCGCATG
                **  ****** *******  ***** **   *****   ******  ** **********

Dsuz_tub        TATGTTAATACTGT
Dmel_tub        TATGTAAACGCTGT
                ***** **  ****
```

```
Dsuz_tub        ACGCTAAGTCGGAGGACGGACGGTCAGGTACTAGCGGCGGTGTCTAGTTTGCTCTTGCCA
Dsim_tub        ACTCCAAGTCGGAGGATAGTCGATCTAAGACCAGCGCCGGTTCCTAGTTCGCTTGTGCCA
                ** * ***********  * ** **    ** **** ****  ****** ***  *****

Dsuz_tub        TCAACAATGCGTGCCATGCCTTTTCTCGAATGTATTTTA--------CAATTTCTGAAGA
Dsim_tub        GAAGTAACGCGTGCCATGCATCTTCACCAATGTATTTTAATATGTAATATTCTCCAATGA
                  *  ** *********** * *** * ***********         * * **  * **

Dsuz_tub        CGTCGGGATTGGAAATCCCAAAGTATTAATAAGCACATTGTTTATAAGACTCGCATGTAT
Dsim_tub        CGATGGGATTCGAAATCCTTAATTAGTAAAAAGACGATTGTTGGTAAGACTCGCATGTAT
                **  ****** *******  ** ** *** ***   ******  ****************

Dsuz_tub        GTTAATACTGT
Dsim_tub        -TAAACGCTGT
                 * **  ****
```

Alignments for *blw*

```
Dsuz_Blw        ACGCCCTGATCATCTACGACGATTTGTCCAAGCAGGCTGTGGCCTACCGTCAGATGTCCC
Dbia_Blw        ACGCCCTGATCATCTACGACGATTTGTCCAAGCAGGCTGTGGCCTACCGTCAGATGTCCC
                ************************************************************
```

```
Dsuz_Blw        TGCTGCTGCGTCGTCCCCCAGGTCGTGAGGCCTACCCCGGCGATGTGTTCTACCTGCATT
Dbia_Blw        TGCTGCTGCGTCGTCCCCCAGGTCGTGAGGCCTACCCCGGCGATGTGTTCTACCTGCATT
                ************************************************************

Dsuz_Blw        CGCGTCTGCTTGAGCGTGCCGCCAAGATGTCCCCTGCCATGGGAGGTGGCTCCCTGACTG
Dbia_Blw        CGCGTCTGCTGGAGCGTGCCGCCAAGATGTCCCCTGCCATGGGAGGTGGCTCCCTGACTG
                ********** *************************************************

Dsuz_Blw        CCCTGCCCGTGATCGAGACCCAGGCTGGTGATGTGTCCGCCTACATTCCA
Dbia_Blw        CCCTGCCCGTGATCGAGACCCAGGCTGGTGATGTGTCCGCCTACATTCCA
                **************************************************


Dsuz_Blw        ACGCCCTGATCATCTACGACGATTTGTCCAAGCAGGCTGTGGCCTACCGTCAGATGTCCC
Dmel_Blw        ACGCCCTGATCATCTACGATGATTTGTCCAAGCAGGCTGTGGCCTACCGTCAGATGTCCC
                ******************* ****************************************

Dsuz_Blw        TGCTGCTGCGTCGTCCCCCAGGTCGTGAGGCCTACCCCGGCGATGTGTTCTACCTGCATT
Dmel_Blw        TGTTGCTGCGTCGTCCCCCAGGTCGTGAGGCCTACCCCGGTGATGTGTTCTACCTGCATT
                ** ************************************* *******************

Dsuz_Blw        CGCGTCTGCTTGAGCGTGCCGCCAAGATGTCCCCTGCCATGGGAGGTGGCTCCCTGACTG
Dmel_Blw        CGCGTCTGCTGGAGCGTGCCGCCAAGATGTCCCCCGCCATGGGAGGCGGTTCCCTGACTG
                ********** *********************** *********** ** **********

Dsuz_Blw        CCCTGCCCGTGATCGAGACCCAGGCTGGTGATGTGTCCGCCTACATTCCA
Dmel_Blw        CTCTGCCCGTGATCGAGACCCAGGCTGGCGATGTGTCCGCCTACATTCCA
                •   ************************** *********************


Dsuz_Blw        ACGCCCTGATCATCTACGACGATTTGTCCAAGCAGGCTGTGGCCTACCGTCAGATGTCCC
Dsim_Blw        ACGCCCTGATCATCTACGATGATTTGTCCAAGCAGGCTGTGGCTTACCGTCAGATGTCCC
                ******************* *********************** ****************

Dsuz_Blw        TGCTGCTGCGTCGTCCCCCAGGTCGTGAGGCCTACCCCGGCGATGTGTTCTACCTGCATT
Dsim_Blw        TGCTGCTGCGTCGTCCCCCAGGTCGTGAGGCCTATCCCGGTGATGTGTTCTACCTGCATT
                ********************************** ***** *******************

Dsuz_Blw        CGCGTCTGCTTGAGCGTGCCGCCAAGATGTCCCCTGCCATGGGAGGTGGCTCCCTGACTG
Dsim_Blw        CGCGTCTGCTGGAGCGTGCCGCCAAGATGTCCCCCGCCATGGGAGGCGGTTCCCTGACTG
                ********** *********************** *********** ** **********

Dsuz_Blw        CCCTGCCCGTGATCGAGACCCAGGCTGGTGATGTGTCCGCCTACATTCCA
Dsim_Blw        CTCTGCCCGTGATCGAGACCCAGGCTGGCGATGTGTCCGCCTACATTCCA
                * ************************** *********************
```

Alignments for *RpL19*

```
Dsuz_RpL19      ggtctgatcatcaagaagcccgtcgtggtccactcccgctaccgtgtgcgcaagaacacc
Dbia_RpL19      ggtctgatcatcaagaagcccgtcgtggtccactcccgctaccgtgtgcgcaagaacacc
                ************************************************************

Dsuz_RpL19      gaggcgcgccgcaagggccgccactgcggattcggaaagcgcaagggtacggcaaacgcc
Dbia_RpL19      gaggcgcgtcgcaagggccgccactgcggattcggaaagcgcaagggtacggcgaacgcc
                ********.*********************************************.*****

Dsuz_RpL19      cgcatgcccaccaagctggtgtggatgcagcgccagcgcgtcctgcgccgcctgctgaag
Dbia_RpL19      cgtatgcccaccaagctgttgtggatgcagcgccagcgcgtcctgcgccgcctgctgaag
                **.*************** *****************************************

Dsuz_RpL19      aagtaccgcgacagcaagaagatcgacaggcacct
Dbia_RpL19      aagtaccgcgacagcaagaagatcgacaggca---
                ********************************
```

```
Dsuz_RpL19      ggtctgatcatcaagaagcccgtcgtggtccactcccgctaccgtgtgcgcaagaacacc
Dmel_Rpl19      ggtctgatcatcaagaagcccgtcgtggtccactcccgttaccgtgtgcgcaaaaacacc
                **************************************.**************.******

Dsuz_RpL19      gaggcgcgccgcaagggccgccactgcggattcggaaagcgcaagggtacggcaaacgcc
Dmel_Rpl19      gaggcccgccgcaagggacgtcactgcggattcggaaagcgtaagggtactgcgaacgcc
                ***** *********** **.********************.******** **.******

Dsuz_RpL19      cgcatgcccaccaagctggtgtggatgcagcgccagcgcgtcctgcgccgcctgctgaag
Dmel_Rpl19      cgcatgcctaccaagctgctgtggatgcagcgccagcgcgttctgcgccgcctgttgaag
                ********.********* **********************.************.*****

Dsuz_RpL19      aagtaccgcgacagcaagaagatcgacaggcacct
Dmel_Rpl19      aagtaccgcgacagcaagaagattgacaggcacct
                ***********************.***********


Dsuz_RpL19      ggtctgatcatcaagaagcccgtcgtggtccactcccgctaccgtgtgcgcaagaacacc
Dsim_RpL19      ggtctgatcatcaagaagcccgtcgtggtccactcccgttaccgtgtgcgcaaaaacacc
                **************************************.**************.******

Dsuz_RpL19      gaggcgcgccgcaagggccgccactgcggattcggaaagcgcaagggtacggcaaacgcc
Dsim_RpL19      gaggcccgccgcaagggacgtcactgcggattcggaaagcgcaagggtactgcgaacgcc
                ***** *********** **.***************************** **.******

Dsuz_RpL19      cgcatgcccaccaagctggtgtggatgcagcgccagcgcgtcctgcgccgcctgctgaag
Dsim_RpL19      cgtatgcctaccaagctgctgtggatgcagcgccagcgcgttctgcgccgcctgttgaag
                **.*****.********* **********************.************.*****

Dsuz_RpL19      aagtaccgcgacagcaagaagatcgacaggcacct
Dsim_RpL19      aagtaccgcgacagcaagaagattgacaggcacct
                ***********************.***********
```

Alignments for *ace*

```
Dsuz_Ace        tgccacgggacaaatatctcgaaattatgaacaacattttcggcaaggcaacgcaggcgg
Dbia_Ace        tgccacgggacaaatatcttgaaattatgaacaacattttcggcaaggcaaagcaggcgg
                *******************.******************************* ********

Dsuz_Ace        aacgcgaggccattatttccagtacaccagctgggagggcaatccgggataccagaacc
Dbia_Ace        aacgcgaggccatt------cagtacaccagctgggagggcaatccgggataccagaatc
                **************      **************************************.*

Dsuz_Ace        aacagcaaatcggacgtgcggtgggcgatcacttcttcacctgccccaccaacgagtatg
Dbia_Ace        aacagcaaatcggacgcgccgtgggcgaccacttcttcacctgccccaccaacgagtatg
                ****************.** ********.*******************************

Dsuz_Ace        cccaggctctggcggagcgaggcgcctccgtgcactactac
Dbia_Ace        cccaggcactggcggagcgaggcgcctccgtgcactactac
                ******* *********************************


Dsuz_Ace        tgccacgggacaaatatctcgaaattatgaacaacattttcggcaaggcaacgcaggcgg
Dmel_Ace        tgccacgggacaaatacctggaaattatgaacaatatttttggcaaggcaacgcaagcgg
                ****************.** **************.*****.**************.****

Dsuz_Ace        aacgcgaggccattatttccagtacaccagctgggagggcaatccgggataccagaacc
Dmel_Ace        aacgcgaggccatcatttccagtataccagttgggaaggcaatcctggctatcagaacc
                *************.**********.*****.*****.******** ** **.********

Dsuz_Ace        aacagcaaatcggacgtgcggtgggcgatcacttcttcacctgccccaccaacgagtatg
Dmel_Ace        agcagcaaatcggacgcgccgtgggcgatcacttcttcacctgccccaccaacgagtatg
                *.**************.** ****************************************
```

```
Dsuz_Ace        cccaggctctggcggagcgaggcgcctccgtgcactactac
Dmel_Ace        cccaggctctggcggagcgaggcgcttccgtgcactactac
                *************************.***************



Dsuz_Ace        tgccacgggacaaatatctcgaaattatgaacaacattttcggcaaggcaacgcaggcgg
Dsim_Ace        tgccacgggacaaatacctggaaattatgaacaacattttcggcaaggcaacgcaagcgg
                ****************.** ***********************************.****

Dsuz_Ace        aacgcgaggccattattttccagtacaccagctgggagggcaatccgggataccagaacc
Dsim_Ace        aacgcgaggccatcattttccagtataccagttgggaaggcaatcctggctatcagaacc
                *************.***********.*****.*****.******** ** **.*******

Dsuz_Ace        aacagcaaatcggacgtgcggtgggcgatcacttcttcacctgccccaccaacgagtatg
Dsim_Ace        agcagcaaatcggacgcgccgtgggcgatcacttcttcacctgccccaccaacgagtatg
                *.**************.** ****************************************

Dsuz_Ace        cccaggctctggcggagcgaggcgcctccgtgcactactac
Dsim_Ace        cccaggctctggcggagcgaggcgcctccgtgcattactac
                **********************************.******
```

Supplementary Figure S2: *D. suzukii* target gene fragments

Name: y-Tubulin 23C
Symbol: *yTub23C*
Molecular function: Structural constituent of cytoskeleton
Length: 183 bp
Spotted wing Flybase accession: DS10_00003610
```
>Dsuz_tub
ACGCTAAGTCGGAGGACGGACGGTCAGGTACTAGCGGCGGTGTCTAGTTTGCTCTTGCCA
TCAACAATGCGTGCCATGCCTTTTCTCGAATGTATTTTACAATTTCTGAAGACGTCGGGA
TTGGAAATCCCAAAGTATTAATAAGCACATTGTTTATAAGACTCGCATGTATGTTAATAC
TGT
```

Name: Vacuolar H^+-ATPase 26kD subunit
Symbol: *Vha26*
Molecular function: Proton transporting ATPase activity
Length: 222 bp
Spotted wing Flybase accession: DS10_00010885
```
>Dsuz_vha26
ACGTGGCCACTCAGCATTGTTGCCCGCCGAAACACCGAAATGGTCCCAAAAAACCAATTTCGCTGCTTCGA
GGGCAACGGACACGTGTGCAGCTGCCACTGGCCCATCAAAAGCCCCGAAAGTCATCAATGTCTGTTGTTGA
GTTAGCGAAAGTAACGAATACACACTATTAAGCACGCAACATTTACTCTCCATAATTCACCGCACCCAAGA
CGAAGAAGT
```

Name: Ribosomal protein L19
Symbol: *RpL19*
Molecular function: Structural constituent of ribosome
Length: 215 bp
Spotted Wing Flybase accession: DS10_00003610
```
>Dsuz_Rpl19
GGTCTGATCATCAAGAAGCCCGTCGTGGTCCACTCCCGCTACCGTGTGCGCAAGAACACC
GAGGCGCGCCGCAAGGGCCGCCACTGCGGATTCGGAAAGCGCAAGGGTACGGCAAACGCC
CGCATGCCCACCAAGCTGGTGTGGATGCAGCGCCAGCGCGTCCTGCGCCGCCTGCTGAAG
AAGTACCGCGACAGCAAGAAGATCGACAGGCACCT
```

Name: Acetylcholine esterase
Symbol: *Ace*
Molecular function: Acetylcholine esterase activity
Length: 221 bp
Spotted Wing Flybase accession: DS10_00680001
```
>Dsuz_Ace
TGCCACGGGACAAATATCTCGAAATTATGAACAACATTTTCGGCAAGGCAACGCAGGCGG
AACGCGAGGCCATTATTTTCCAGTACACCAGCTGGGAGGGCAATCCGGGATACCAGAACC
AACAGCAAATCGGACGTGCGGTGGGCGATCACTTCTTCACCTGCCCCACCAACGAGTATG
CCCAGGCTCTGGCGGAGCGAGGCGCCTCCGTGCACTACTAC
```

Name: Bellwether
Symbol: *blw*
Molecular function: proton-transporting ATPase activity
Length: 230 bp
Spotted Wing Flybase accession: DS10_00002141

```
>Dsuz_Blw
ACGCCCTGATCATCTACGACGATTTGTCCAAGCAGGCTGTGGCCTACCGTCAGATGTCCC
TGCTGCTGCGTCGTCCCCCAGGTCGTGAGGCCTACCCCGGCGATGTGTTCTACCTGCATT
CGCGTCTGCTTGAGCGTGCCGCCAAGATGTCCCCTGCCATGGGAGGTGGCTCCCTGACTG
CCCTGCCCGTGATCGAGACCCAGGCTGGTGATGTGTCCGCCTACATTCCA
```
